# Effects of Lacosamide Treatment on Epileptogenesis, Neuronal Damage and Behavioral Comorbidities in a Rat Model of Temporal Lobe Epilepsy

**DOI:** 10.3390/ijms22094667

**Published:** 2021-04-28

**Authors:** Michaela Shishmanova-Doseva, Dimitrinka Atanasova, Yordanka Uzunova, Lyubka Yoanidu, Lyudmil Peychev, Pencho Marinov, Jana Tchekalarova

**Affiliations:** 1 Department of Pharmacology Toxicology and Pharmacotherapy, Medical University-Plovdiv, 4002 Plovdiv, Bulgaria; peych@propolisbg.com; 2 Institute of Neurobiology, Bulgarian Academy of Sciences (BAS), 1113 Sofia, Bulgaria; didiatanasova7@gmail.com; 3 Department of Anatomy, Faculty of Medicine, Trakia University, 6003 Stara Zagora, Bulgaria; 4 Department of Bioorganic Chemistry, Medical University-Plovdiv, 4002 Plovdiv, Bulgaria; d_anny@abv.bg (Y.U.); lubka.yoanidu@gmail.com (L.Y.); 5 Institute of Information and Communication Technologies, BAS, 1113 Sofia, Bulgaria; pencho.marinov@gmail.com

**Keywords:** lacosamide, pilocarpine, oxidative stress, inflammation, neuronal loss, hippocampus

## Abstract

Clinically, temporal lobe epilepsy (TLE) is the most prevalent type of partial epilepsy and often accompanied by various comorbidities. The present study aimed to evaluate the effects of chronic treatment with the antiepileptic drug (AED) lacosamide (LCM) on spontaneous motor seizures (SMS), behavioral comorbidities, oxidative stress, neuroinflammation, and neuronal damage in a model of TLE. Vehicle/LCM treatment (30 mg/kg, p.o.) was administered 3 h after the pilocarpine-induced status epilepticus (SE) and continued for up to 12 weeks in Wistar rats. Our study showed that LCM attenuated the number of SMS and corrected comorbid to epilepsy impaired motor activity, anxiety, memory, and alleviated depressive-like responses measured in the elevated plus maze, object recognition test, radial arm maze test, and sucrose preference test, respectively. This AED suppressed oxidative stress through increased superoxide dismutase activity and glutathione levels, and alleviated catalase activity and lipid peroxidation in the hippocampus. Lacosamide treatment after SE mitigated the increased levels of IL-1β and TNF-α in the hippocampus and exerted strong neuroprotection both in the dorsal and ventral hippocampus, basolateral amygdala, and partially in the piriform cortex. Our results suggest that the antioxidant, anti-inflammatory, and neuroprotective activity of LCM is an important prerequisite for its anticonvulsant and beneficial effects on SE-induced behavioral comorbidities.

## 1. Introduction

Epilepsy is one of the most common neurological disorders, widespread all over the world, and affecting more than 50 million people of different ages, sex, race, and social class [1]. Temporal lobe epilepsy (TLE) is a clinically prevalent type of partial epilepsy. Severe head injury, stroke, intracranial infections, brain tumors, or preceding status epilepticus (SE) are factors that can induce functional and chemical changes in the brain and development of epilepsy [2,3,4,5]. Due to alterations occurring during epileptogenesis, patients tend to develop various comorbidities such as cognitive and psychiatric disorders, which have a detrimental impact on their quality of life [6].

Animal models of human complex partial seizures, provoked by chemoconvulsants such as pilocarpine (Pilo) or kainic acid (KA), are characterized by epileptogenesis after SE, a subsequent chronic period with spontaneous motor seizures (SMS), and comorbid psychiatric responses [4,7,8]. The pathological changes that are triggered in the first hours after SE can cause initial damage and severe widespread neuronal loss in different brain areas, including the hippocampus, endopiriform nucleus, and piriform cortex [9]. The hippocampus has a pivotal role in learning, memory, and mood control, and since it is highly susceptible to injury, these functions can be easily impaired in patients with TLE [10].

Research evidence has recently outlined oxidative stress as a crucial factor responsible for cognitive dysfunction associated with TLE [3,11,12]. It is defined as an imbalance created by overproduction of free radicals and the diminished capacity of the antioxidant systems to neutralize them in the brain. The generation of excessive reactive oxygen (ROS) and nitrogen (RNS) species leads to disrupted mitochondrial function and secondary damage to DNA, proteins, and lipids [12,13,14,15,16]. These changes in major cellular components have been established as an underlying cause of learning and memory impairment, especially when the hippocampus is subjected to injury either due to direct exposure to oxidative stress, or subsequently induced neuroinflammation [7,17].

Experimental and clinical data have suggested a close relationship between epileptogenesis and neuroinflammation due to increased release of the major pro-inflammatory cytokines as tumor necrosis factor-α (TNF-α) and interleukin-1β (IL-1β) which are generated by the activated glial cells [18]. Permanently activated inflammatory pathways can trigger long-term deleterious effects in neurotransmission and hyperexcitability of the CNS in the chronic epileptic tissue [19,20]. Besides, the pro-inflammatory cytokines modulate neuronal plasticity and long-term potentiation (LTP) which impairs cognitive abilities such as learning and memory [21,22,23,24,25,26,27]. 

Despite the vast number of new antiepileptic drugs (AEDs) in the last ten years, there is still little information on whether they can produce a disease-modifying effect during the latent period, or if the applied therapy has been only symptomatic. In clinical trials, administration of conventional AEDs, such as valproic acid, carbamazepine, and phenytoin, has failed to prevent the processes of epileptogenesis [28]. Lacosamide (LCM) is a third-generation new AED with a novel and unique mechanism of action. The drug regulates the long-term availability of sodium channels by selectively enhancing their slow inactivation and, in this manner, controls the pathophysiologic neuronal hyperexcitability [29]. 

To date, only some preclinical data give evidence that LCM exerts beneficial effects on oxidative stress and inflammatory reaction in other models. Moreover, there is insufficient data on the beneficial influence of this AED on concomitant-to-epileptogenesis devastating effects on cellular homeostasis. Also, whether the anticonvulsant activity is involved in the effects of LCM on comorbid behavioral responses in epilepsy requires further clarification. Therefore, the present study aimed to explore the effect of LCM on deleterious consequences in a Pilo-induced post-SE rat model of TLE. The effect of chronic LCM treatment during epileptogenesis on spontaneous motor seizures (SMS) was also evaluated. We propose that the AED LCM exerts anticonvulsant and beneficial effects on behavioral comorbidities in epilepsy via ameliorating activity against oxidative stress and neuronal loss in the brain. 

## 2. Results

### 2.1. Seizure Activity in Rats Treated with Pilo

With the exception of 2 rats, 58 rats developed SE following Pilo injection. Sixteen rats (27%) died during the SE. The survivals were equally allocated to two treatment groups (Pilo–veh, Pilo–LCM, *n* = 21 per group). 

**Figure 1:**
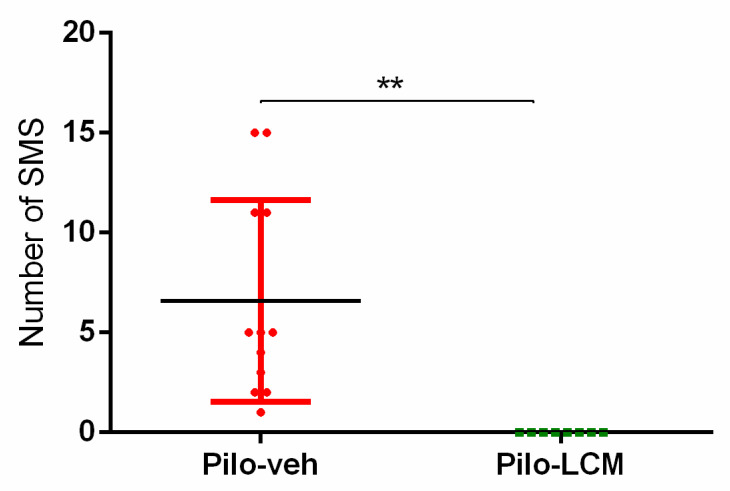
Effect of chronic treatment with lacosamide (LCM) on the number of spontaneous motor seizures (SMS) in a pilocarpine-induced model of temporal lobe epilepsy. ** *p* < 0.01 compared to the Pilo–veh group.

In total, 16 rats were continuously video-monitored for a week starting 60 days after SE, a period characterized by a stable amount of SMS. The Pilo–veh group showed higher seizure frequency when the lights were on (Mann–Whitney U test, *p* < 0.01) (Figure 1). All the Pilo–veh treated rats showed SMS (total number of SMS: 61; mean ± S.E: 6.6 ± 1.5, median 5 seizures/days, *n* = 8). Chronic treatment with LCM significantly alleviated the frequency of SMS (Pilo–LCM group: —total number of SMSs: 0; mean ± S.E: 0 ± 0; median 0 seizures/days, *n* = 8) (Student *t*-test: t = 3.651; *p* ≤ 0.01 Pilo–LCM compared to the Pilo–veh group).

### 2.2. Behavioral Tests

#### 2.2.1. Activity Cage

**Figure 2:**
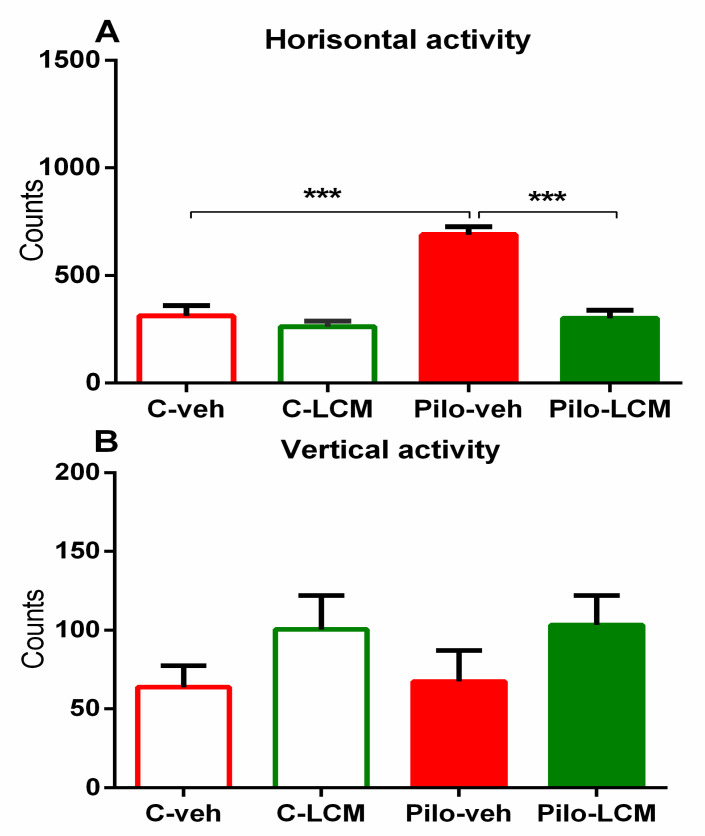
Effect of chronic treatment with LCM on (**A**) horizontal activity and (**B**) vertical activity in activity cage in naïve rats and epileptic animals. *** *p* < 0.01 compared to the Pilo–veh group.

There was a significant main effect of Epilepsy (F(1, 44) = 29.857, *p* < 0.001), LCM treatment (F(1, 44) = 32.863, *p* < 0.001), and significant Epilepsy × LCM treatment interaction (F(1, 44) = 19.294, *p* < 0.001) on the number of horizontal movements. The post-hoc test revealed that the Pilo–veh rats showed an elevated number of horizontal movements (*p* < 0.001 compared to C–veh group) (Figure 2A). Two-way ANOVA revealed that neither LCM treatment nor Epilepsy demonstrated a significant effect on the number of vertical movements. (Figure 2B). Chronic treatment with LCM per se did not influence ambulation in control conditions, while it was able to alleviate the Pilo-induced hyperactivity (*p* < 0.001 compared to Pilo–veh group).

#### 2.2.2. Elevated Plus Maze (EPM) Test

**Figure 3:**
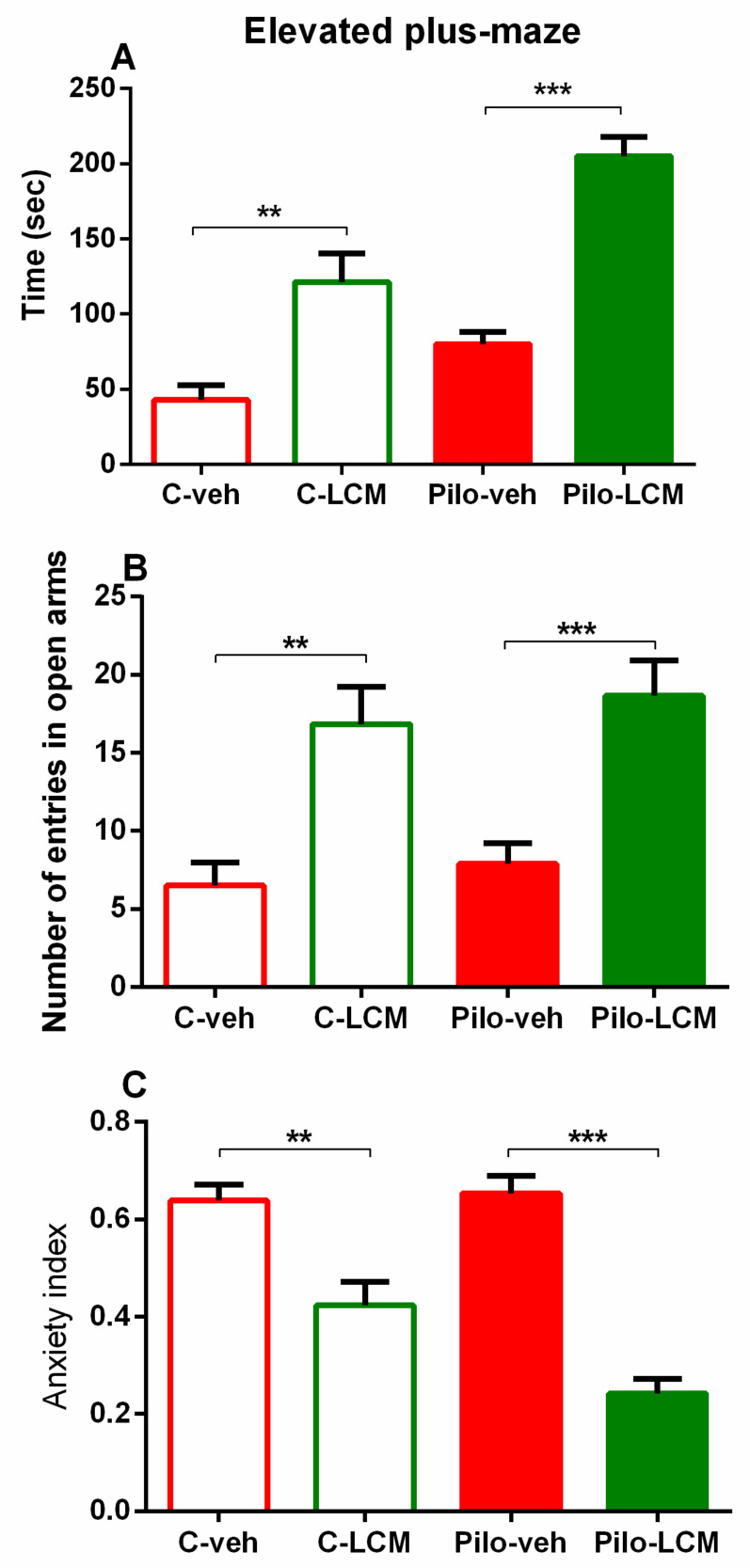
Effect of chronic treatment with LCM on (**A**) time (sec) in the open arms in the elevated plus maze test (**B**) number of entries in the open arms and (**C**) the Anxiety index in naïve and epileptic animals. ** *p* < 0.01 C–LCM vs. C–veh group; *** *p* < 0.001 Pilo–LCM vs. Pilo–veh group.

There was a significant main effect of Epilepsy (F(1, 44) = 20.548, *p* < 0.001) and LCM treatment (F(1, 44) = 58.153, *p* < 0.001) on the time spent in the open arms of the EPM. The post-hoc test demonstrated that both the C–LCM group and the Pilo–LCM group had a significantly longer time in the aversive area compared to the C–veh group (*p* < 0.01, *p* < 0.001, respectively) (Figure 3A). The analysis of variance revealed a significant main effect of LCM treatment on the number of entries into the open arms of the EPM (F(1, 44) = 30.492, *p* < 0.001). The post-hoc test showed that both groups treated with LCM had higher numbers of entries into the open arms (*p* < 0.01 C–LCM compared to C–veh group; *p* < 0.001 Pilo–LCM compared to Pilo–veh group) (Figure 3B). A lower anxiety index was demonstrated for the groups treated with LCM: Epilepsy (F(1, 44) = 4.839, *p* = 0.05), LCM treatment (F(1, 44) = 69.131, *p* < 0.001), and Epilepsy × LCM treatment interaction (F(1, 44) = 6.701, *p* < 0.05). The post-hoc test revealed that both LCM-treated groups had a decreased anxiety index (*p* < 0.01 C–LCM compared to C–veh group; *p* < 0.001 Pilo–LCM compared to Pilo–veh group) (Figure 3C). 

#### 2.2.3. Sucrose Preference Test (SPT)

**Figure 4:**
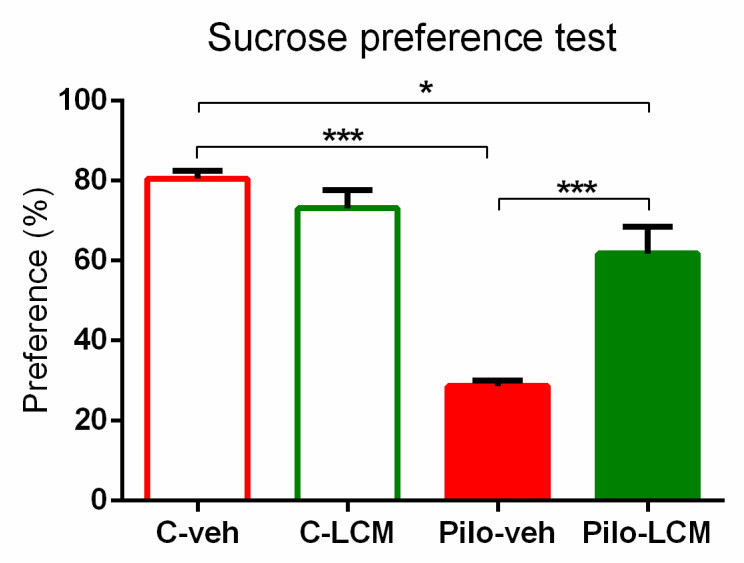
Effect of chronic treatment with LCM on sucrose preference test (%) in naïve rats and epileptic animals. * *p* < 0.05 Pilo–LCM vs. C–veh group; *** *p* < 0.001 Pilo–veh vs. C–veh group; *** *p* < 0.001 Pilo–LCM vs. Pilo–veh group.

For the SPT, the analysis of variance showed significant main effects of Epilepsy (F(1, 44) = 55.231, *p* < 0.001) and LCM treatment (F(1, 44) = 9.069, *p* = 0.01) as well as interaction between them (F(1, 44) = 23.015, *p* < 0.001). The post-hoc test revealed depressive-like behaviour in the Pilo–veh group when compared to controls (*p* < 0.001), while LCM treatment during epileptogenesis partially managed to reverse it (*p* < 0.001, Pilo–LCM compared to Pilo–veh group; *p* < 0.05 Pilo–LCM compared to C–veh) (Figure 4).

#### 2.2.4. Object Recognition Test (ORT)

**Figure 5:**
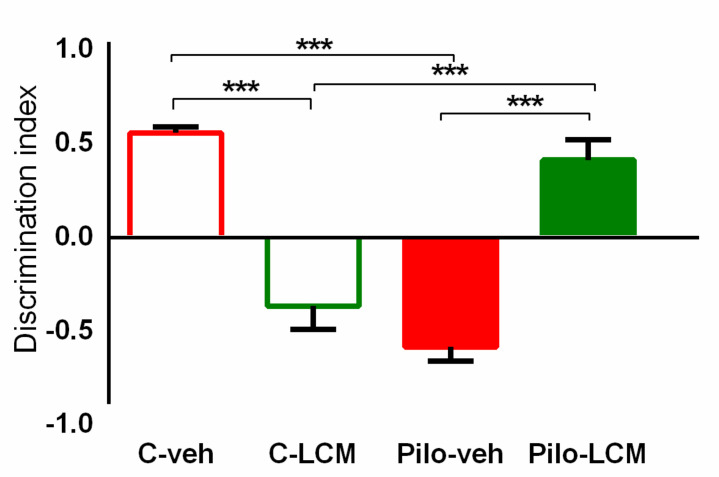
Effect of chronic treatment with LCM on the discrimination index in the object recognition test in naïve and epileptic animals. *** *p* < 0.001 C–LCM vs. C–veh group; ***p < 0.001 Pilo–veh vs. C–veh group; *** *p* < 0.001 Pilo–LCM vs. Pilo–veh group and *** *p* < 0.001 Pilo–LCM vs. C–LCM group.

Two-way ANOVA revealed the Epilepsy × LCM treatment interaction (F(1, 44) = 109.536, *p* < 0.001) for the discrimination index. The post-hoc test demonstrated that the Pilo–veh and C–LCM group had impaired object recognition memory (*p* < 0.001 compared to C–veh group) while the long-term LCM treatment during epileptogenesis managed to diminish the negative influence of epilepsy and increase the discrimination index (DI) (*p* < 0.001 compared to C–LCM and Pilo–veh group, respectively) (Figure 5).

#### 2.2.5. Radial Arm Maze Test (RAM)

**Figure 6:**
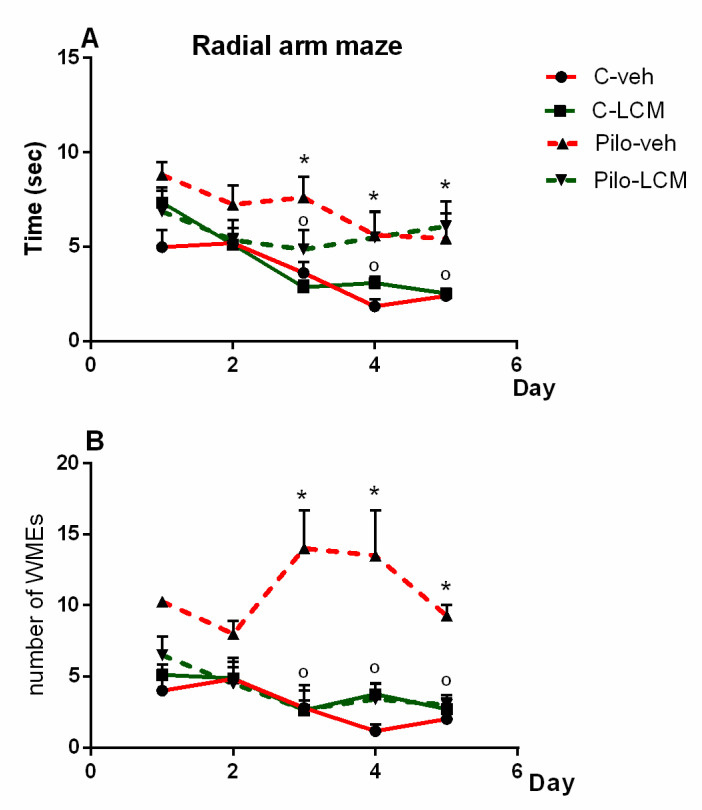
Effect of chronic treatment with LCM (**A**) the time (sec) needed to fulfill the task in the radial arm maze test and (**B**) on the number of working memory errors (WMEs) in naïve and epileptic animas. * *p* < 0.05 Pilo–veh vs. C–veh group; *p* < 0.05 Pilo–LCM vs. Pilo–veh group.

For the number of working memory errors (WMEs), three-way ANOVA showed a significant main effect of Epilepsy (F(1, 160) = 11.526, *p* < 0.001) and LCM treatment (F(1, 160) = 4.162, *p* = 0.044) as well as with Epilepsy × Treatment interaction (F(1, 160) = 9.623, *p* < 0.002). For the time needed to fulfill the task, analysis of variance demonstrated a main effect of Epilepsy (F(1, 160) = 9.013, *p* = 0.003) and Time (F(4, 160) = 8.262, *p* < 0.001). The post-hoc test confirmed that the Pilo–veh group exhibited more WMEs during the last three sessions of RAM test compared to the C–veh group (*p* < 0.05) while the treatment with LCM alleviated the SE-induced memory impairment by shortening the time needed to fulfill the task at sessions three, four, and five and decreasing the number of WMEs, respectively, in the RAM test (*p* < 0.05 compared to Pilo–veh group) (Figure 6A,B).

### 2.3. Markers of Oxidative Stress

#### 2.3.1. SOD Activity Assay

**Figure 7:**
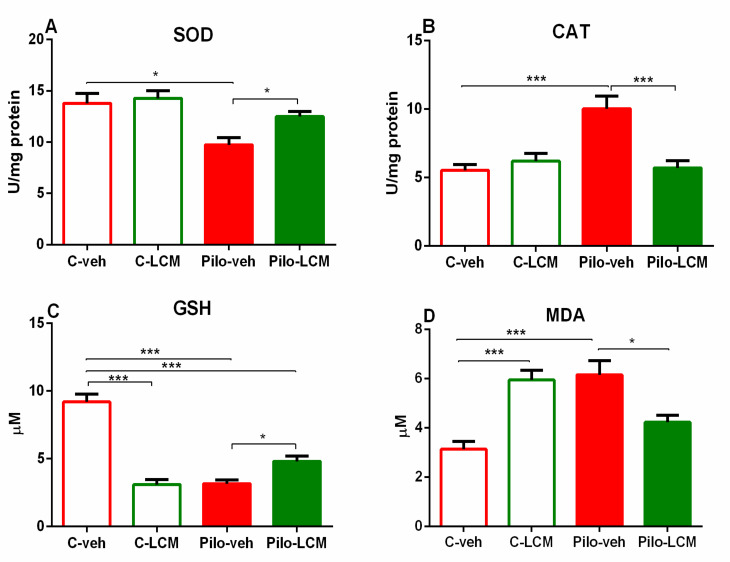
Effect of chronic treatment with LCM (**A**) on SOD activity (U/mg protein) in rat hippocampus of naïve and epileptic animals. * *p* < 0.05 Pilo–veh vs. C–veh group; * *p* < 0.05 Pilo–LCM vs. Pilo–veh group; (**B**) on Catalase (CAT) activity (U/mg protein) in rat hippocampus of naïve and epileptic animals. *** *p* < 0.001 C–LCM vs. C–veh group; Pilo–veh vs. C–veh group; *** *p* < 0.001 Pilo–LCM vs. Pilo–veh group; (**C**) on reduced glutathione (GSH) levels (µM) in rat hippocampus of naïve and epileptic animals. *** *p* < 0.001 Pilo–veh vs. C–veh group; *** *p* < 0.001 Pilo–LCM vs. C–veh group; * *p* < 0.05 Pilo–LCM vs. Pilo–veh group; (**D**) on malondialdehyde (MDA) levels (µM) in rat hippocampus of naïve and epileptic animals. *** *p* < 0.001 Pilo–veh vs. C–veh group; *** *p* < 0.001 C–LCM vs. C–veh group; * *p* < 0.05 Pilo–LCM vs. Pilo–veh group.

Two-way ANOVA demonstrated a significant main effect of Epilepsy (F(1, 28) = 11.164, *p* < 0.01) on SOD activity in the hippocampus. The Pilo–veh group decreased the SOD activity in comparison with the C–veh group (*p* < 0.05). While the C–LCM group did not change SOD activity in control conditions, the treatment with this AED restored the Pilo-induced diminished enzyme activity (*p* < 0.05 compared to Pilo–veh group) (Figure 7A). 

#### 2.3.2. CAT Activity Assay

The main effect of Epilepsy (F(1, 28) = 10.019, *p* < 0.01), LCM treatment (F(1, 28) = 8.288, *p* < 0.01), and interaction between both factors (F(1, 28) = 15.660, *p* < 0.001) was detected on the CAT activity in the hippocampus. The post-hoc test demonstrated that the Pilo–veh group significantly increased the CAT activity compared to the control group (*p* < 0.001) (Figure 7B). In contrast, a decrease in the CAT activity was detected for the Pilo–LCM group (*p* < 0.001 compared to Pilo–veh group).

#### 2.3.3. GSH Assay

Analysis of variance revealed significant main effects of Epilepsy (F(1, 28) = 26.430, *p* < 0.001), LCM treatment (F(1, 28) = 28.043, *p* = 0.001), and interaction between both factors (F(1, 28) = 84.811, *p* < 0.001) on GSH level in the hippocampus. The post-hoc test showed that the Pilo–veh group significantly decreased GSH levels in comparison with the controls (*p* < 0.001) (Figure 7C). The treatment with LCM per se also diminished the levels of GSH (*p* < 0.001 compared to C–veh group). Although the Pilo–LCM group significantly mitigated the Pilo-induced diminishing effect on GSH levels, the beneficial effect of LCM treatment during epileptogenesis was incomplete (*p* < 0.05 compared to Pilo–veh group, *p* < 0.001 compared to C–veh group).

#### 2.3.4. MDA Levels

Two-way ANOVA demonstrated a significant Epilepsy × LCM treatment interaction (F(1, 28) = 33.681, *p* < 0.001). The post-hoc test showed that the Pilo–veh and C–LCM groups significantly elevated the MDA levels compared to the C–veh group (*p* < 0.001) while the chronic LCM treatment during epileptogenesis alleviated the lipid peroxidation (*p* < 0.05 Pilo–LCM compared to Pilo–veh group) (Figure 7D).

### 2.4. IL-1β Levels and TNF-α Levels

**Figure 8:**
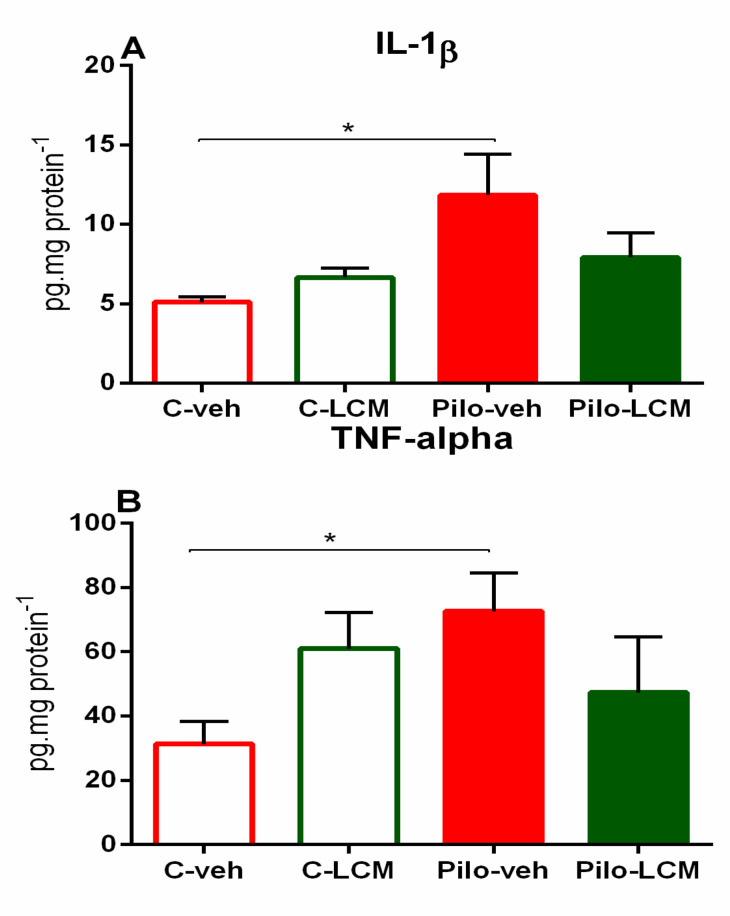
Effect of chronic treatment with LCM (**A**) on IL-1β levels (pg.mg protein-1) and (**B**) on TNF-α levels (pg.mg protein-1) in rat hippocampus of naïve and epileptic animals. * *p* < 0.05 Pilo–veh vs. C–veh group.

Two-way ANOVA demonstrated a main Epilepsy effect (F(1, 28) = 6.351, *p* = 0.02) for the IL-1β levels without either LCM treatment effect or Epilepsy × LCM interaction (*p* > 0.05). The post-hoc test confirmed that the Pilo–veh group significantly elevated IL-1β levels compared to the matched control group (*p* = 0.02), while the chronic LCM treatment partially alleviated the Pilo-induced elevation of the pro-inflammatory cytokine in the hippocampus (*p* > 0.05 Pilo–LCM compared to Pilo–veh) (Figure 8A). 

**Figure 9:**
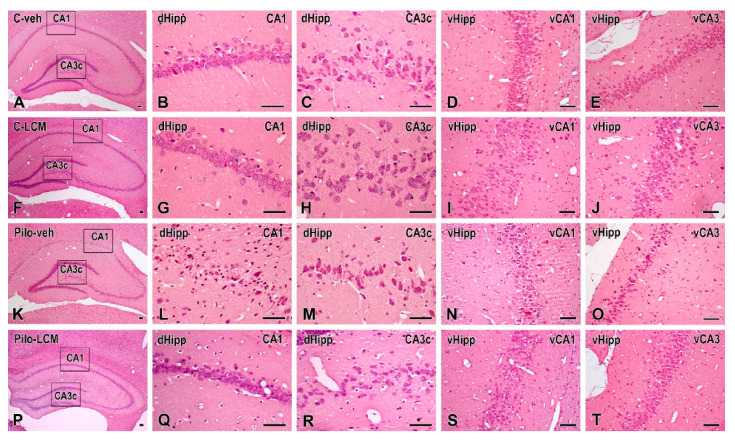
Representative hematoxylin and eosin stained coronal sections of the dorsal (dHipp) and ventral (vHipp) hippocampal formation in (**A**–**E**) vehicle-treated control rats (C–veh), (**F**–**J**) control rats treated with lacosamide (C–LCM), (**K**–**O**) pilocarpine (Pilo)-induced epileptic rats (Pilo–veh) and (**P**–**T**) pilocarpine epileptic rats treated with lacosamide (Pilo–LCM). The illustrative microphotographs in the second and third columns are higher magnifications of the boxed areas of the images in the first column from the CA1 and CA3c areas of the dHipp, respectively. Please note that the Pilo–veh rats (**K**) showed severe neuronal loss in the CA1 (**L**) and CA3c (**M**) pyramidal cell layers in the dHipp and in the ventral hippocampal CA1 (vCA1) (**N**) and vCA3 (**O**) neurons when compared to the control rats (**A**–**E**). Overview of dHipp (**P**), higher magnifications in the dorsal CA1 (**Q**), dorsal CA3c (**R**) and overview of vCA1 (**S**) and vCA3 (**T**) reveal a partial restoration of neurons in Pilo–LCM-treated rats. Scale bar = 50 μm.

ANOVA of variance revealed a main Epilepsy effect (F(1, 28) = 4.857, *p* = 0.039) without an LCM treatment effect as well as Epilepsy × LCM interaction (*p* > 0.05) on TNF-α levels. The post-hoc test demonstrated that the Pilo–veh group significantly increased the TNF-α levels compared to the control group in the hippocampus (*p* < 0.018) (Figure 9B). The treatment with LCM after SE partially suppressed the elevated TNF-α levels (*p* > 0.05 Pilo–LCM compared to Pilo–veh) (Figure 8B).

### 2.5. Histology

**Figure 10:**
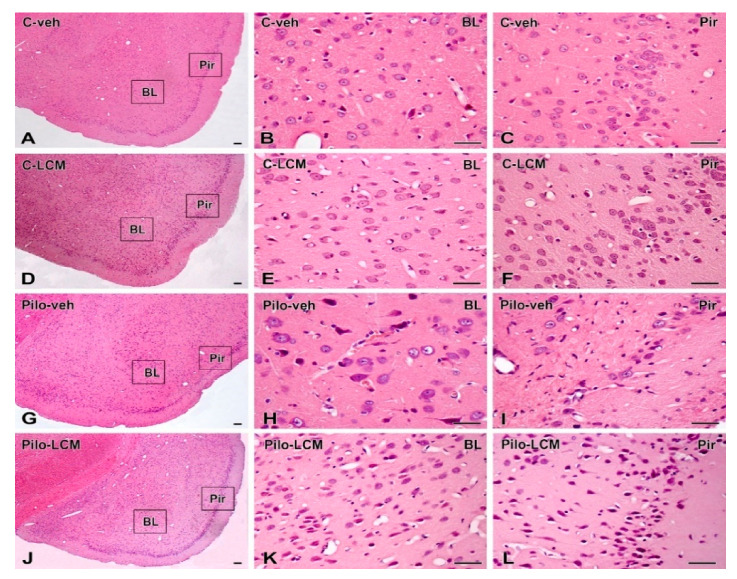
Morphological effects of lacosamide on the piriform cortex and basolateral amygdala in rats. (**A**) A representative conventionally H&E-stained section at the level of the basolateral amygdaloid nucleus (BL) and piriform cortex (Pir) in vehicle-treated control rats (C–veh). Higher magnification of the box areas of the image in (**A**) showing the normal morphology and density of neuronal population in BL (**B**) and Pir (**C**) in control rats. (**D**) Low-resolution view of the BL and Pir in lacosamide-treated control animals (C–LCM). (**E**,**F**) show higher magnification of these areas, respectively. (**G**) Overview of the same area shown in (**A**,**D**) in pilocarpine (Pilo)-induced epileptic rats treated with vehicle (Pilo–veh). (**H**,**I**) Higher magnifications demonstrating neuronal loss in the basolateral nucleus of amygdala (BL) and piriform cortex (Pir). Low (**J**) and high (**K**,**L**) magnification of the BL and Pir in lacosamide-treated epileptic rats (Pilo–LCM) and depiction of the protective effect of lacosamide on neuronal survival. Scale bar = 50 μm.

Representative cresyl violet-stained sections from the hippocampus (dorsal, dHipp and ventral, vHipp) as well as from the piriform cortex (Pir) and basolateral amygdala (BL) of the control groups (C–veh and C–LCM) and epileptic groups (Pilo–veh and Pilo–LCM) are presented in Figure 9A–T and Figure 10A–L, respectively. Statistical data from two-way ANOVA followed by a post-hoc Bonferroni, if a significance was detected, are demonstrated in Table 1. Except for the ventral hippocampus, all brain structures were analyzed into the septal, septo-temporal, and temporal sections.

**Figure 11:**
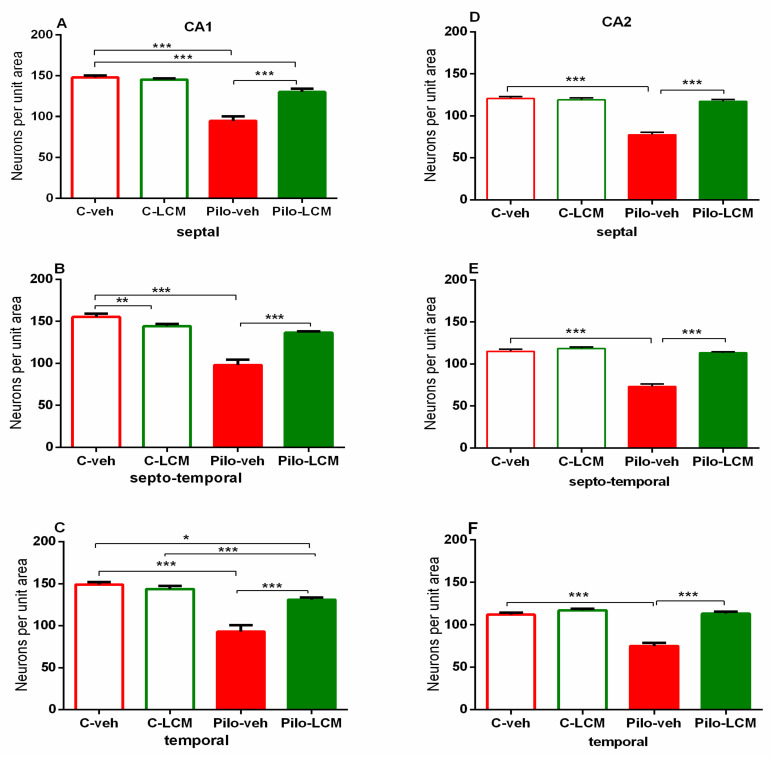
Effect of chronic treatment with LCM in naïve and epileptic animals on the histology scores. Neuronal damage in the dorsal hippocampus in the (**A**) septal, (**B**) septo-temporal and (**C**) temporal CA1 region, (**D**) septal, (**E**) septo-temporal and (**F**) temporal CA2 region. * *p* < 0.05 Pilo–LCM vs. C–veh; ** *p* < 0.01 LCM-veh vs. C–veh; *** *p* < 0.001 Pilo–veh vs. C–veh group; *** *p* < 0.001 Pilo–LCM vs. C–veh group; *** *p* < 0.001 Pilo–LCM vs. Pilo–veh group.

**Figure 12:**
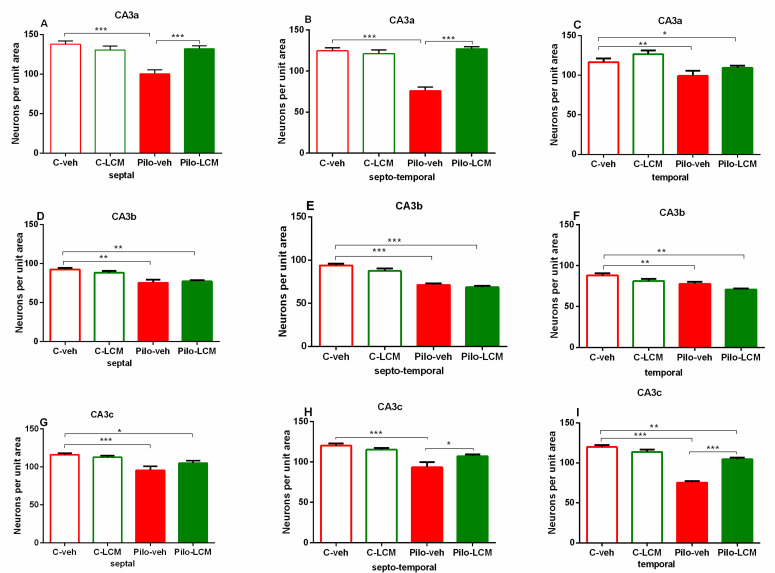
Effect of chronic treatment with LCM in naïve and epileptic animals on the histology scores. Neuronal damage in the dorsal hippocampus in the CA3a (septal (**A**), septo-temporal (**B**), and temporal (**C**)), CA3b (septal (**D**), septo-temporal (**E**), and temporal (**F**)) and CA3c (septal (**G**), septo-temporal (**H**), and temporal (**I**)) region. ** *p* < 0.01, *** *p* < 0.001 Pilo–veh vs. C–veh group; * *p* < 0.05, *** *p* < 0.001 Pilo–LCM vs. Pilo–veh group; * *p* < 0.05, ** *p* < 0.01, *** *p* < 0.001 Pilo–LCM vs. C–veh group.

**Figure 13:**
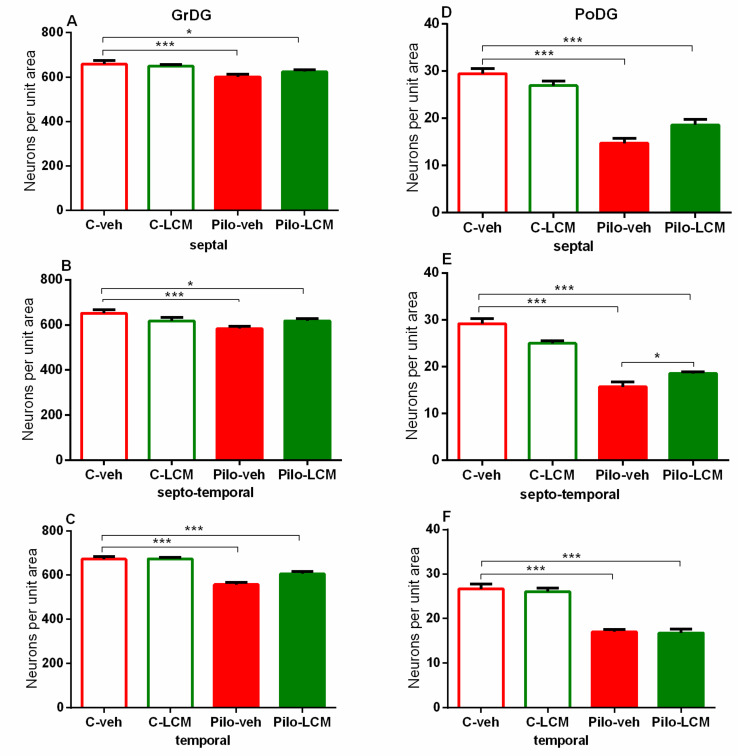
Effect of chronic treatment with LCM in naïve and epileptic animals on the histology scores. Neuronal damage in the GrDG (septal (**A**), septo-temporal (**B**), and temporal (**C**)) and PoDG (septal (**D**), septo-temporal (**E**), and temporal (**F**)). *** *p* < 0.001 Pilo–veh vs. C–veh group; * *p* < 0.05, *** *p* < 0.001 Pilo–LCM vs. C–veh group; * *p* < 0.05 Pilo–LCM vs. Pilo–veh group.

For the dorsal hippocampus, the post-hoc test confirmed that Pilo in vehicle-treated rats caused neuronal damage in the septal, septo-temporal, and temporal CA1, CA2 (Figure 11A–F), as well as the CA3a, CA3b, and CA3c, region (*p* < 0.01 and *p* < 0.001, respectively) (Figure 12A–I). The LCM treatment exerted a strong protective effect in the CA1-CA2 region (septal, septo-temporal, and temporal) (*p* < 0.001 compared to Pilo–veh group) though in the septal and temporal CA1 region the protection against Pilo-induced neuronal loss was incomplete (*p* < 0.001 and *p* < 0.05 compared to C–veh group) (Figure 11A, C). Area-specific alleviation of neuronal damage was exerted by LCM in the CA3 region with a significant and stronger effect in the CA3a (septal and septo-temporal) and CA3c (septo-temporal), partial in the CA3c (temporal) (*p* < 0.05 and *p* < 0.001 compared to Pilo–veh group; *p* < 0.01 Pilo–LCM compared to C–veh) and lack of protection in the CA3a (temporal), CA3b (septal, septo-temporal and temporal), and CA3c (septal) (*p* < 0.05, *p* < 0.01, *p* < 0.001 Pilo–LCM compared to C–veh) (Figure 12A–I). A profound neuronal damage was also detected in the dentate gyrus (DG) (septal, temporal and temporal Granular (Gr)DG and (Polymorphic) PoDG) (*p* < 0.001 compared to C–veh) (Figure 13A–F). The treatment with LCM after Pilo-induced SE did not exert neuroprotection in the DG (*p* < 0.001 compared to C–veh). With the exception of the partial effect in the septo-temporal PoDG (*p* < 0.05 Pilo–LCM compared to Pilo–veh, *p* < 0.001 Pilo–LCM compared to Pilo–LCM compared to C–veh), LCM was unable to protect epileptic rats from the Pilo-induced neuronal loss in the GrDG (*p* < 0.001 Pilo–veh compared to C–veh group; *p* < 0.05 and *p* < 0.001 Pilo–LCM compared to C–veh group) and PoDG (septal, temporal) (*p* < 0.001 compared to C–veh group) (Figure 13A–F).

**Figure 14:**
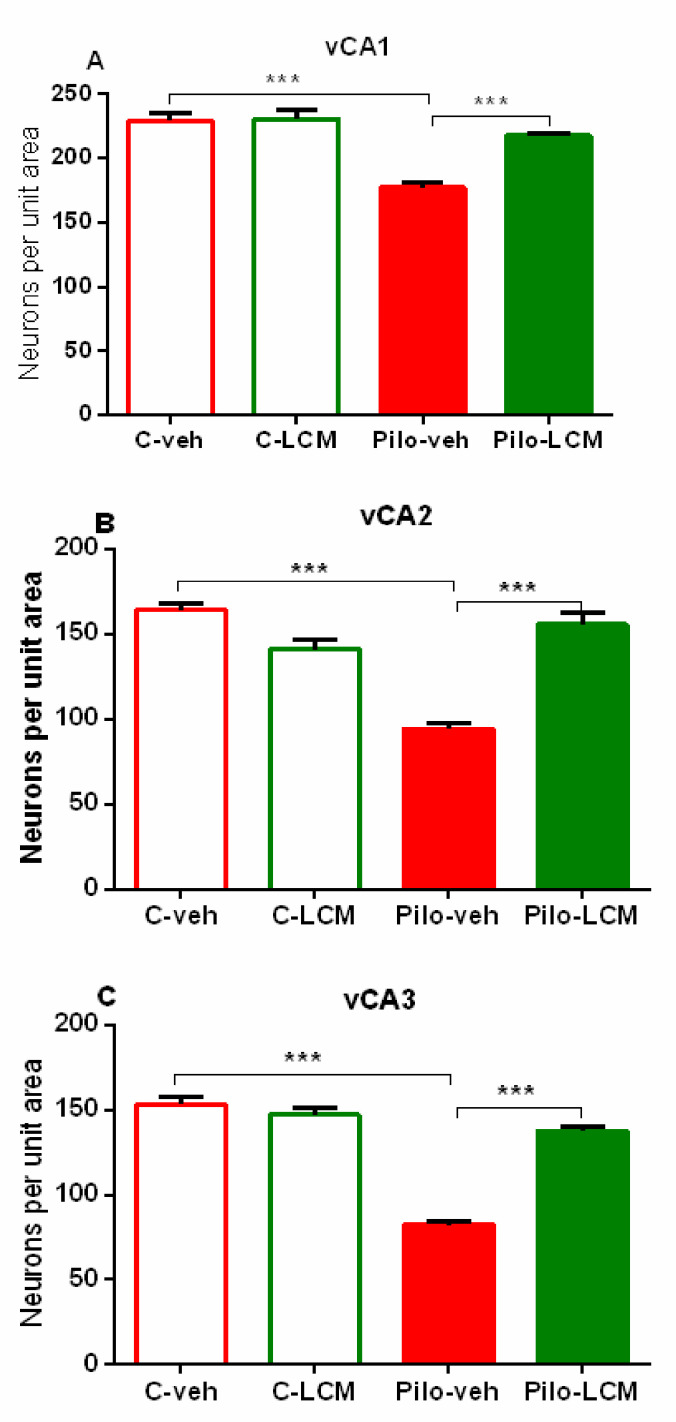
Effect of chronic treatment with LCM in naïve and epileptic animals on the histology scores. Neuronal damage in the ventral hippocampus (**A**) CA1 region, (**B**) CA2 region, and (**C**) CA3 region. *** *p* < 0.001 Pilo–veh vs. C–veh group; *** *p* < 0.001 Pilo–LCM vs. Pilo–veh group.

Treatment with LCM showed a strong neuroprotective effect in the three areas CA1, CA2, and CA3 of the ventral hippocampus (*p* < 0.001 compared to Pilo–veh group) (Figure 14A–C).

**Figure 15:**
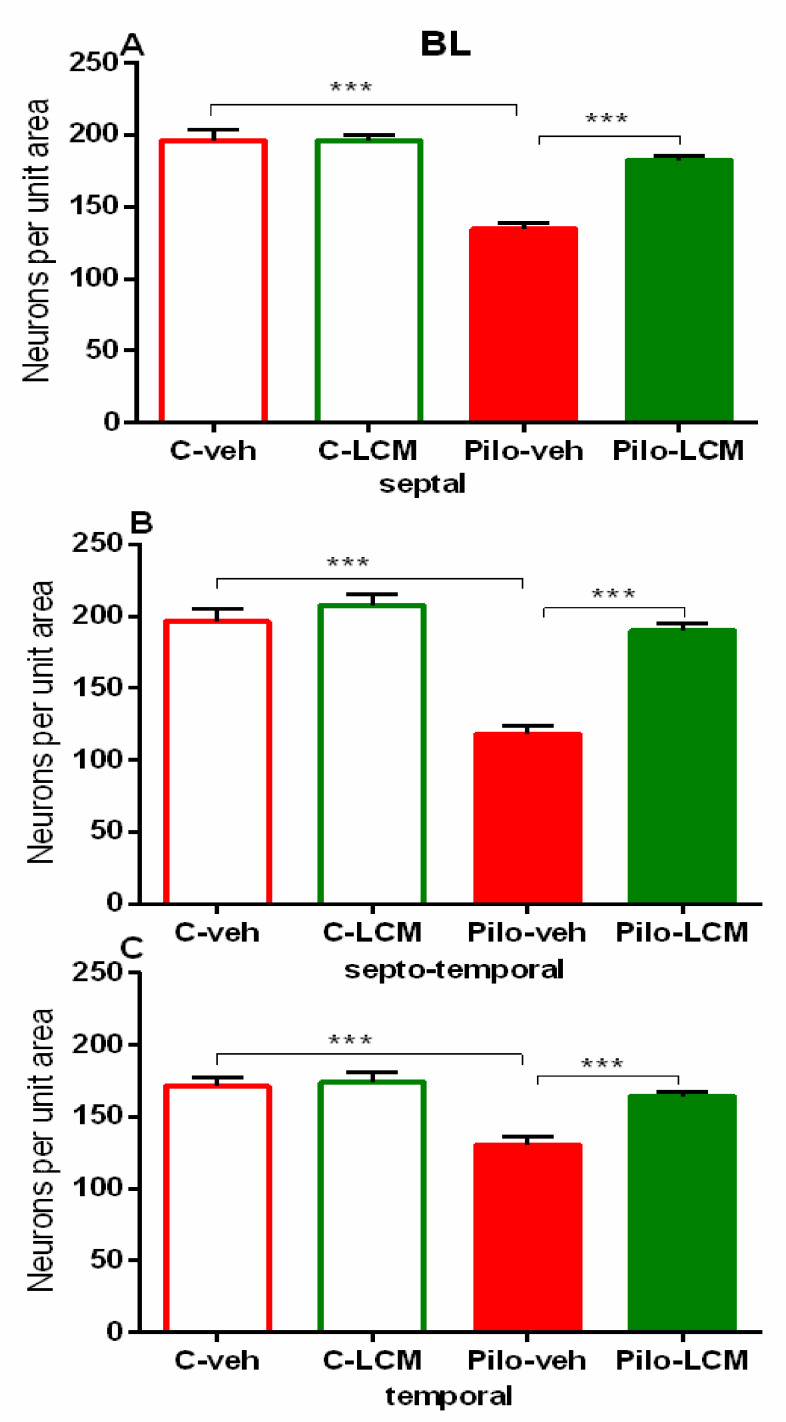
Effect of chronic treatment with LCM in naïve and epileptic animals on the histology scores. Neuronal damage in the basolateral amygdaloid nucleus (**A**) septal, (**B**) septo-temporal, and (**C**) temporal. *** *p* < 0.001 Pilo–veh vs. C–veh group; *** *p* < 0.001 Pilo–LCM vs. Pilo–veh group.

**Figure 16:**
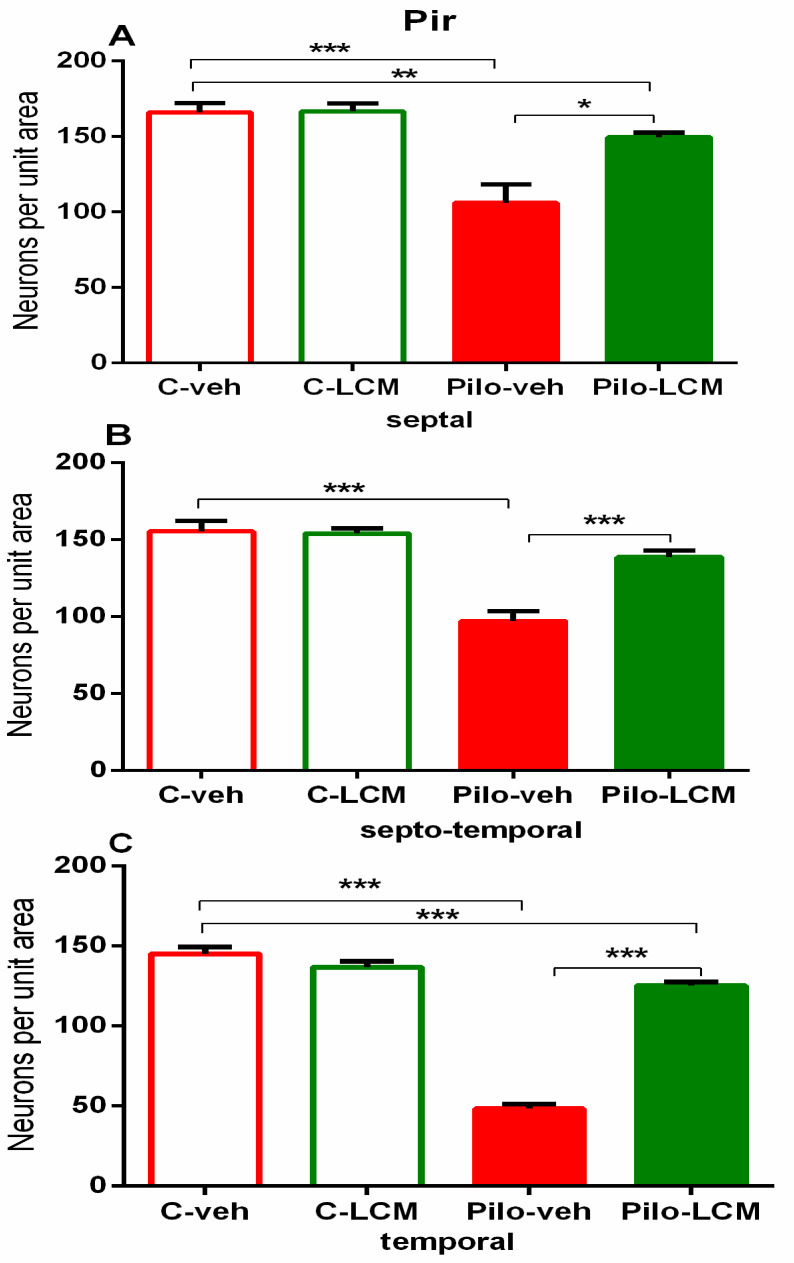
Effect of chronic treatment with LCM in naïve and epileptic animals on the histology scores. Neuronal damage in the piriform cortex (**A**) septal, (**B**) septo-temporal, and (**C**) temporal. *** *p* < 0.001 Pilo–veh vs. C–veh group; * *p* < 0.05, *** *p* < 0.001 Pilo–LCM vs. Pilo–veh group; ** *p* < 0.01, *** *p* < 0.001 Pilo–LCM vs. C–veh group.

Neuronal damage in the BL and Pir as a result of Pilo-induced epileptogenesis was profound and detected in the septal, septo-temporal, and temporal area (*p* < 0.001 compared to C–veh group) (Figure 15A–C and Figure 16A–C, respectively). The LCM treatment after SE exerted strong protection against neuronal damage in the BL (septal, septo-temporal, and temporal: *p* < 0.001 compared to Pilo–veh group). This beneficial influence was also evident in the septo-temporal Pir (*p* < 0.001 Pilo–LCM compared to Pilo–veh) while it was incomplete in the septal and temporal Pir (septal: *p* = 0.002 Pilo–LCM compared to C–veh, *p* = 0.013 Pilo–LCM compared to Pilo–veh group; temporal: *p* < 0.001 Pilo–LCM compared to C–veh and Pilo–veh) (Figure 16A–C). 

## 3. Discussion

In the present study, a comprehensive exploration of the new AED LCM used for the treatment of seizures was conducted both in naїve and pilocarpine-treated rats in a chronic epileptic state. We report that while LCM per se showed some deleterious behavioral responses in the age-matched controls, it exerted anxiolytic, antidepressant, and memory-improving effects in epileptic rats, suggesting a true “disease-dependent” activity. The beneficial influence of this AED chronically administered during epileptogenesis on comorbid behavioral disturbance should be due to its direct anticonvulsant effect and closely related to its antioxidant activity in the hippocampus, its neuroprotective effect in several severely damaged brain areas, namely, the dorsal, ventral hippocampus and basolateral amygdala, as well as a partial anti-inflammatory effect. 

In agreement with numerous literature data from animal models, chronic treatment with AEDs during epileptogenesis suppressed SMS in the pilocarpine rat model [30,31,32]. The present results confirm a previous report according to which LCM can exert neuroprotection in brain structures which are susceptible to seizures by suppressing the hippocampal mossy fiber sprouting in KA-induced SE [33]. Recently, we have found that repeated treatment with LCM during KA-induced SE, used at the same dose as in the present work, suppresses epileptiform activity (EEG and video-recorded) in the chronic epileptic phase [34]. However, the anti-epileptogenic effect of this AED reported in Pilo and KA-induced post-SE models was accompanied by model- and region-specific neuroprotection. Thus, while complete protection was detected only in the dorsal CA1c (temporal) region of the hippocampus after five injections with LCM during KA-induced SE, chronic treatment with LCM up to three months after Pilo-induced SE produced a profound activity against Pilo-induced seizures and neuronal loss in the dorsal hippocampus (CA1, septo-temporal, CA2, CA3a (septal, septo-temporal), CA3c (septo-temporal), the whole ventral hippocampus, and the BL and Pir (septo-temporal). Although model-related divergence in the effect of LCM on neuronal damage cannot be ruled out, our findings suggest that long-term administration during epileptogenesis is more effective and crucial treatment protocol than short-term protocol during SE. The anticonvulsant effect of LCM detected in both models of TLE seems not to be a prerequisite for the effect of this AED against seizure-evoked neuronal loss in limbic structures. However, this protection might contribute to the ameliorating effect of this drug on comorbid psychiatric responses. We report that LCM succeeded in protecting both the dorsal and ventral hippocampus from the Pilo-induced neuronal loss. It is well-known that the two parts of the hippocampus, dorsal and ventral, have not only different excitability and seizure susceptibility [35,36], but also functionality. While the dorsal hippocampus is responsible for cognition, the ventral hippocampus is associated with emotions [37]. Besides, damage to structures such as BL and Pir might worsen epileptic symptoms and cause cognitive dysfunction [38]. Cognitive and mood impairments are one of the most frequent comorbidities for people suffering from epilepsy. These problems could be associated with the disease neuropathology, seizures, and side effects of the applied drug therapy. Multiple cognitive domains in these patients could be affected, such as attention, working memory, short- and long-term memory, and executive functions [39]. The findings of the present study show that LCM impairs object recognition memory in naïve animals, while long-term treatment with LCM during epileptogenesis manages to diminish the negative influence of epilepsy and facilitates memory consolidation and retrieval. This task determines the spontaneous tendency of rodents to explore novel objects in preference to a familiar one. This preference is linked to several brain regions, including the dorsal hippocampus [40,41]. In the reward-based cognitive task, we aimed to evaluate the spatial memory performance over a five-day testing period. Chronic treatment with LCM in rats applied after induction of recurrent seizures managed to improve the hippocampal-dependent spatial memory by decreasing the number of errors and time to fulfill the task. Taken together, these results suggest beneficial effects of LCM on cognitive outcomes in epileptic animals. Still, little is known about the effects of this new AED on cognitive performance. Clinical data have shown that LCM can have beneficial effects on executive functions when compared with carbamazepine, and its long-term cognitive effects are comparable with lamotrigine and superior to those of topiramate [42,43]. Experimental studies suggest that in naïve rats, LCM can have a detrimental effect on attention, spatial learning, and memory [44,45], which is consistent with our current and previous data demonstrating impairment of active and passive learning and memory, as well as recognition memory in naïve animals [46]. 

Mood alterations such as anxiety and depression, which are present with anhedonia and despair-like behavior, are of high prevalence among people with epilepsy [47]. In the present study, while there was no increase in the anxiety index in the pilo–veh group, long-term treatment with LCM has anxiolytic properties in naïve rats and in animals with TLE, as it managed to increase the time spent, the number of entries, and the anxiety index in the open arm of the EPM, which is an aversive area. Moreover, LCM managed to alleviate the symptoms of depression in the epileptic animals during the post-SE period and to alleviate epilepsy-induced hyperactivity in the activity cage. Data about the effect of LCM on anxiety levels and depression are very scarce, although experimental as well as clinical studies have recently shown improved symptoms after acute or chronic treatment with this drug in rats and epileptic patients [47,48,49,50], as well as in patients with bipolar disorder without epilepsy [51]. 

The mechanisms involved in the epileptogenic processes leading to behavioral comorbidities such as cognitive disabilities, anxiety, and depression are still not well elucidated. There is a growing number of experimental and clinical evidence supporting the hypothesis that oxidative stress plays an important role in both the initiation and progression of epilepsy. It can additionally contribute to neuronal hyperexcitability and cell loss through numerous mechanisms, including oxidative damage to membrane proteins such as neurotransmitter receptors and ion channels [17,52,53,54]. Scientific data suggest that brain damage is mainly caused by lipid peroxidation, and can be determined by measuring MDA levels, which is a major end product of the interaction between free radicals and unsaturated fatty acids [55]. 

In the present study, epileptic animals showed increased oxidative stress with significantly elevated MDA levels, diminished SOD activity, and decreased GSH levels in the hippocampus, which agrees with other studies [12,56,57]. LCM treatment during epileptogenesis alleviated the lipid peroxidation, while, in naïve animals, the drug exacerbated this process in the hippocampus. LCM managed to ameliorate the SOD activity in both epileptic and naïve rats. In compliance with other authors, we have demonstrated that hippocampal catalase activity increased after Pilo-induced SE as cellular protection against the increased ROS production [57,58]. Treatment with LCM in naïve and epileptic animals managed to restore its activity to control levels.

The levels of the endogenous antioxidant GSH were also ameliorated by the drug treatment during the recurrent seizures. Our results are in agreement with a previous study which has demonstrated that LCM decreased the formation of free radicals 48 h after SE induced by Pilo, since the authors reported reduced MDA levels and increased GSH levels [59]. Moreover, recent studies have revealed the antioxidant properties of LCM in an experimental model of traumatic spinal cord injury and lipopolysaccharide-induced neuroinflammation through increasing the levels of total antioxidant status in different brain areas such as the hippocampus, cortex, and cerebellum [60,61]. Chronic oxidative stress is also associated with cognitive dysfunction, therefore, the decrease of the levels of free radicals caused by LCM could have long-term effects and be responsible for the seizure attenuation and better cognitive performance of the animals treated with it during epileptogenesis, thus producing a disease-modifying effect such as preventing the occurrence of anxiety and depressive-like symptoms. However, unlike the beneficial effect of LCM treatment in epileptic rats, it decreased the GSH levels and accompanied lipid peroxidation in chronically treated naïve rats. This deleterious effect on oxidative stress correlated with the observed deleterious effect on object recognition memory.

The presence of excessive production of oxygen free radicals promotes the development and persistence of inflammation that affects epileptogenesis through different mechanisms, including lower seizure threshold, blood–brain barrier dysfunction, neuronal damage, and increased neuronal excitability [57,62,63]. The literature data have demonstrated that pro-inflammatory cytokines such as TNF-α and IL-1β can affect the hippocampal synaptic and molecular plasticity, which in turn reduces the neurogenesis and LTP. This process plays a pivotal role for memory consolidation and hippocampal-dependent learning and memory, and indirectly accelerates the adverse cognitive consequences [24,26,38,64,65,66,67]. In the present study, we have found a significant increase in the levels of TNF-α and IL-1β in the epileptic group, which is in agreement with other studies presenting rapid and persistent neuroinflammatory responses [56,57,68]. Long-term treatment with LCM was able to decrease their levels during the period of recurrent seizures and to produce a mild anti-inflammatory effect in the hippocampus. Our results are in agreement with recent experimental studies which demonstrated decreased levels of these pro-inflammatory cytokines caused by LCM in models of lipopolysaccharide-induced neuroinflammation and paclitaxel-induced peripheral neuropathy [61,69].

## 4. Materials and Methods

### 4.1. Drugs and Reagents

Lacosamide (Vimpat, USB Pharma, Brussels, Belgium); pilocarpine (Sigma Aldrich, Hamburg, Germany); scopolamine methyl nitrate (Sigma Aldrich, Hamburg, Germany); diazepam (Sopharma, Sofia, Bulgaria); IL-1 beta Rat ELISA Kit-Invitrogen-Thermo Fisher Scientific and TNF alpha Rat ELISA Kit-Invitrogen-Thermo Fisher Scientific. Phenazine methosulfate (PMS), nitroblue tetrazolium (NBT), trichloroacetic acid (TCA), thiobarbituric acid (TBA), 5,5′-dithio-bis-(2-nitrobenzoic acid) (DTNB), paraformaldehyde, hematoxylin & eosin (FOT Ltd., Sofia, Bulgaria).

### 4.2. Animals

Male Wistar rats (150–180 g) were obtained from the Animal Center of Medical University–Plovdiv (*n* = 102). Rats were housed in plastic cages (5–6 per cage) and were kept under standardized conditions 12/12h light/dark cycle and controlled temperature (22 ± 1 °C, 50–60% relative humidity). All animals had free access to food and water. This study was performed in strict accordance with the guidelines of the European Community Council directives 86/609/EEC. 0.2010/63/EC. Experiments were approved by the Bulgarian Food Safety Agency No. 206/01.10.2018 and by the Ethical Committee on Human and Animal Experimentation of Medical University–Plovdiv No. 1/28.02.2019.

### 4.3. Study Design, Induction of Status Epilepticus and Treatment with LCM

**Figure 17:**
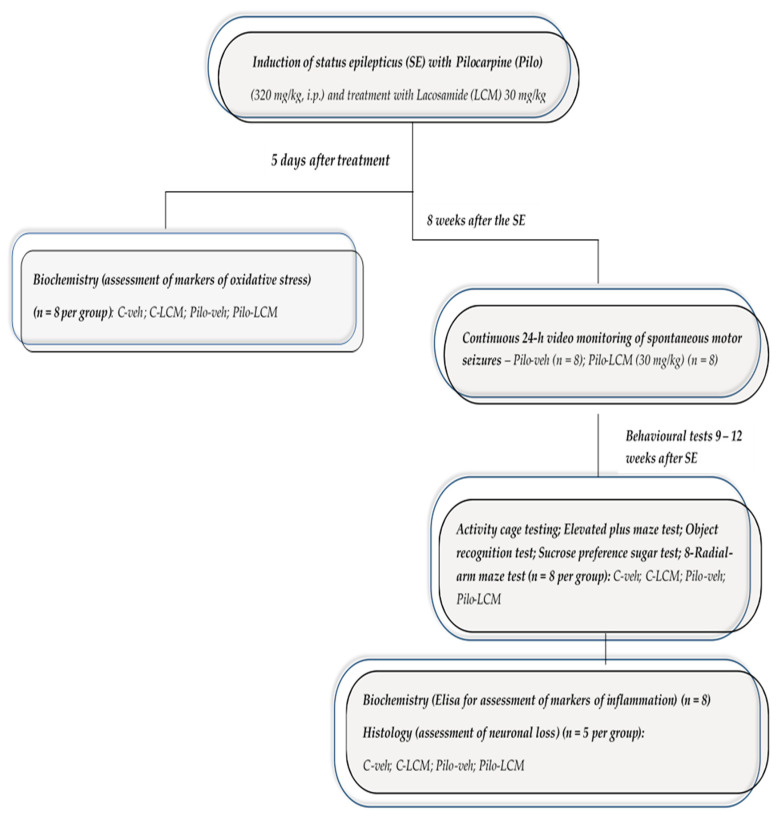
A layout of the study design.

The experimental design is demonstrated in Figure 17. The rats were randomly divided into 4 groups (*n* = 21 each) as follows (1) C–veh (controls treated with saline 1 mL/kg p.os); (2) Pilo–veh (rats injected with Pilo 320 mg/kg i.p. and treated with saline 1 mL/kg p.os); (3) C–LCM (rats treated with LCM 30 mg/kg p.os); (4) Pilo–LCM (rats injected with Pilo 320 mg/kg i.p. and treated with LCM 30 mg/kg p.os). The rats from groups 2 and 4 were injected with Pilo 320 mg/kg i.p., 30 min after the injection of scopolamine methyl nitrate 1 mg/kg i.p., applied to reduce the peripheral cholinergic effects. After Pilo administration, the animals were placed in separate cages, and seizure intensity was assessed based on the Racine scale [70]: Stage 1—mouth and facial movements; Stage 2—head nodding; Stage 3—forelimb clonus; Stage 4—seizures characterized by rearing and continued forelimb clonus; Stage 5—seizures characterized by rearing, forelimb clonus, and falling. Status epilepticus is defined as a single epileptic seizure lasting more than 5 min or two or more seizures within a 5-min-period without returning to normal behavior between them. The criterion for reaching SE was a state with sustained motor seizures (>9 motor seizures/h) at stage 4/5 lasting for at least 2 h, and only these animals were used in further experiments. After 2 h of ongoing seizures, 10 mg/kg diazepam was injected intraperitoneally (i.p), to relieve convulsions and reduce mortality, although some of the animals developed severe seizures (above stage 5). Approximately 3 h after the Pilo injection, the rats were injected subcutaneously (s.c.) with NaCl 0.9% and glucose 5% in equal volumes (up to 3% of body weight) to restore the volume lost. Treatment with veh/LCM, given orally by gavage, started 3 h after the onset of SE. Matched groups were treated with vehicle. Five days after SE, eight animals from each group were decapitated to evaluate the markers of oxidative stress. The rest of the animals were treated for up to 12 weeks. Eight weeks after Pilo injection, all rats were subjected to a battery of tests. At the end of the experiment, all animals were decapitated to evaluate the inflammation (*n* = 8) and the neuronal loss (*n* = 5).

### 4.4. Video Monitoring of Spontaneous Motor Seizures

A continuous video-monitoring (24 h/day for 8 days), executed through an infrared-sensitive camera (S-2016, AVTECH, Taiwan, no. AVC307R) connected to a computer under controlled conditions (12 h/12 h light/dark cycle, light “on” from 08:00 a.m. until 08:00 p.m.) in a separate room, started 8 weeks after SE. Pilo-treated rats were placed in individual, transparent labeled cages. The video recordings were manually assessed off-line, and SMS were scored on the same scale as that used for SE (i.e., stage 3, 4, and 5).

### 4.5. Behavioral Tests

#### 4.5.1. Activity Cage

The activity cage apparatus (Biological Research Apparatus, Ugo Basile, Italy) was a clear plastic box 40 cm^2^ with 40-cm high walls. A printer automatically recorded the number of movements in two categories, horizontal and vertical, captured by the infrared sensor array located on either side of the cage. The animals were individually tracked for 180 s in each session, which were conducted under identical conditions. This testing was performed to measure the spontaneous locomotor activity.

#### 4.5.2. Elevated Plus Maze Test

The apparatus consisted of two open arms (50 × 10 cm^2^), two enclosed arms (50 × 10 × 50 cm^3^), and a central platform (10 × 10 cm^2^) elevated 50 cm above the floor. The rats were placed on the central square of the maze facing an open arm and were allowed 5 min to freely explore the maze. The calculated measures were: (1) time (sec) spent in the open arms; (2) number of entries in the open arms; (3) anxiety index = 1 − ((open arms time/total time) + (number of entries in open arms/total number of entries)/2). The alleys of the maze were cleaned with ethanol solution (60% volume) after the removal of each rat.

#### 4.5.3. Object Recognition Test

The object recognition test consisted of a training session (T1) and a test session (T2). The object recognition test was performed in the open field apparatus (50 × 50 × 50 cm^3^), with objects made of plastic or glass. On the first day, rats were habituated to the OF for 15 min and 24 h later the ORT was performed. During T1, each rat was placed into the arena and exposed to two identical objects (A1 and A2) for 5 min. The objects were placed in a symmetrical position about 10 cm away from the wall. The rats were then moved to their home cage for a 60 min inter-trial interval. To avoid the olfactory trials, the objects and the apparatus were cleaned with 70% ethanol after each rat. Then one of the objects was replaced with a novel one. In the T2, rats were exposed to the familiar object (A1) and the novel object (B) for 5 min. Exploration was defined as directing the nose to the object at a distance of no more than 2 cm and/or touching the object with whiskers/nose and licking. Sitting on the object was not considered as exploratory behavior. The exploration time (sec) for each object in each trial was recorded, and the following measurements were analyzed: total time spent exploring the two objects in the T1 and the DI, which is defined by the difference in exploration time between the novel and familiar objects, divided by the total time spent exploring these two objects in the discrimination phase: (TB − TA)**/**(TB + TA).

#### 4.5.4. Sucrose Preference Test

Sucrose preference was assessed using single-housed rats for three days applying a two-bottle choice test. On the first day, they were habituated to drink from two identical bottles, both of which contained 200 mL of water. On day two, rats were trained in the sucrose preference procedure in which regular water in one of the bottles was replaced with 1% sucrose solution. On day three (test day), the rats were allowed to drink freely from both bottles for 24 h. The experiment was performed for 24 h starting at 08:00 a.m. The position of the bottles was switched daily to avoid position preference. The bottles were refilled each day at the same time in the morning with freshly prepared solutions and water. The bottles were weighed after the test day, and the sucrose consumption and preference (defined as (weight of sucrose ingested)**/**(weight of water ingested + weight of sucrose ingested) × 100) were calculated for each rat.

#### 4.5.5. Radial Arm Maze Test

The hippocampus-dependent spatial memory was evaluated with a RAM test on a stainless steel eight-arm radial maze (Harvard Biosci. Comp., Holliston, Massachusetts, USA) according to a protocol used in a previous study [71,72]. Before the test, rats were put on a diet to reduce their body weight by at least 15%. The diet continued until the last session of the RAM test. A pretest (shaping), executed for three days, started on day six of the diet. The test contained five sessions performed every day after the shaping procedure. Each arm was baited with a piece of a pellet. The session stopped when all pellets were eaten or after a 10-min period. The evaluated parameters were the number of WMEs (re-entry into an arm from which the food pellet had already been retrieved) and the time to fulfill the criteria (with all pellets eaten).

### 4.6. Markers of Oxidative Stress

#### 4.6.1. Preparation of Tissue Homogenate

Hippocampus was homogenized in an ice bath with phosphate buffer (pH = 7), and tissue: buffer ratio 1:25. The homogenate was then centrifuged at 10,000× *g* for 5 min at 4 °C. The obtained supernatant was transferred into another test tube and used for the analysis of CAT and SOD activities, as well as for determining the concentrations of MDA and GSH.

#### 4.6.2. Protein Content

Protein content was determined by a method which employs the use of bicinchoninic acid as a reagent and bovine serum albumin as a standard. A calibration curve was built for the interval 25–1000 µg/mL and protein concentration was calculated according to it. To 2 mL reagent, 100 µL supernatant was added and the mixture was incubated for 30 min at 37 °C. Absorbance was measured against a blank sample at 562 nm. All spectrophotometric measurements were conducted on a CARY 1 spectrophotometer (Varian, Victoria, Australia).

#### 4.6.3. SOD Activity Assay

SOD activity was measured based on the method of Kakar et al. [73]. The reaction mixture was composed of 600 µL 0.052 mM phosphate buffer (pH = 8.16), 50 µL PMS (186 µM), 150 µL, NBT (300 µM), and 150 µL supernatant. The reaction was started by the addition of 100 µL NADH (780 µM) and was stopped after 1 min with 500 µL glacial acetic acid. Absorbance was then measured against a blank sample, at 560 nm. SOD activity was calculated as U/mg protein.

#### 4.6.4. GSH Assay

GSH was measured according to Ellman’s assay. To 50 µL of the homogenate we added 50 µL of 10% TCA. The mixture was centrifuged at 1500× *g* for 15 min and the supernatant was taken for analysis. To 200 µL, 262 mM Tris-HCl (pH = 8.9), 20 µL DTNB, and 50 µL of the supernatant were added. Absorbance was measured after 15 min reaction time, against a blank sample at 412 nm.

#### 4.6.5. CAT Activity Assay

CAT activity was measured using 0.036% solution of hydrogen peroxide in 50 mM phosphate buffer (pH = 7). 100 µL of the supernatant was added to 2.9 mL of the hydrogen peroxide solution and the changes in absorbance were monitored for 3 min at 240 nm. CAT activity was calculated as U/mg protein.

#### 4.6.6. MDA Assay

The reaction mixture composed of 290 µL 0.1M phosphate buffer (pH = 7.4), 100 µL supernatant, 100 µL ascorbic acid (100 µM), and 10 µL iron trichloride (100 µM) was incubated for 1 h at 37 °C. At the end of the incubation time, 500 µL of each 10% TCA and 0.67% TBA were added. Test tubes were then placed in a boiling water bath for 20 min, taken out, placed on ice, and centrifuged at 2500× *g* for 10 min. The absorbance of the supernatant was measured against a blank sample at 535 nm.

### 4.7. Measurement of IL-1 Beta and TNF-Alpha 

After decapitation of the animals, their hippocampi were isolated on ice and preserved at −20 °C until biochemical assay. The tissue samples were homogenized in 10 mL/g tissue in cold buffer containing 10 mM Tris HCL (pH 7.6), 1mM EGTA, 50 mM NaF, 1mM EDTA, and 1mM PMSF. The protein content of each sample was measured by the Bradford method [74]. The measurement of the IL-1 beta and TNF-alpha was executed with invitrogen ELISA kits and the concentration was expressed as pg/mg protein^−1^. 

### 4.8. Histology

Transcardial perfusion was conducted on anesthetized rats (urethane, 1500 mg/kg, i.p.) (*n* = 5) in two steps, with phosphate-buffered saline (0.05 M, pH 7.36) and 4% paraformaldehyde in phosphate buffer (0.1 M, pH 7.3). The carefully isolated brains were post-fixed overnight at 4 °C, embedded in paraffin, and brain slices of 6 μm thick were prepared. Then, they were deparaffinized with xylene, dehydrated with ethanol, and stained with cresyl violet. Counting of intact cells was performed via serial section reconstruction and by using a Nikon Eclipse 80i light microscope (Japan), and a digital camera (Nikon DMX 1200). The analysis of sections was carried out as described previously [34,75,76]. The number of neurons in selected brain areas was calculated by assessing the percentage of stained cells within a regular grid via Nikon’s NIS Elements Digital Imaging software. Three main areas of the dorsal hippocampus (CA1, CA2, and CA3a, CA3b, CA3c), GrGD and the PoDG, BL and the Pir (septal, septo-temporal and temporal) and vHipp (vCA1, vCA2, vCA3) from 18 sections per rat were taken for the counting of neuronal loss.

### 4.9. Statistical Analysis 

Experimental results were presented as mean ± S.E.M. After testing for assumptions of normality of data distribution and homogeneity of variance, two-way ANOVA with factors Epilepsy and Treatment were used for activity cage test; EPM, ORT, SPT. Data of RAM were analyzed by three-way ANOVA. If data were not normally distributed, ANOVA for non-parametric data (Kruskal-Wallis on ranks) followed by the Mann-Whitney U test was used. Post hoc comparisons via the Bonferroni test in case of justification was used. Statistically significant differences were accepted at *p* ≤ 0.05. Student *t*-test was applied for the analysis of SMS data. The analysis was conducted using the SigmaStat^®^ (version 11.0.) statistical package.

## 5. Conclusions

In conclusion, the present study supports the literature and our previous findings that chronic treatment with LCM during epileptogenesis exerts powerful anticonvulsant and beneficial effects against epilepsy-associated behavioral comorbidities. The neuroprotection of this AED in specific limbic regions, as well as its antioxidant and partial anti-inflammatory activity in the hippocampus, must be an important mechanism underlying its anticonvulsant activity in a rat Pilo-induced post-SE model.

## Figures and Tables

**Table 1:** Statistical data for the effect of LCM on neuronal damage (H&E) in the dorsal hippocampus (CA1a, CA1b, CA1c, CA2, CA3a, CA3b, CA3c), Hilus of dentate gyrus (DG), DG, ventral hippocampus (CA1, CA2, CA3), basolateral amygdala and piriform cortex. Two-way analysis of variance was performed followed by a post hoc Bonferroni or Holm Sidak *t*-test when appropriate.

CA1. Septal	Epilepsy [F_1,88_ = 72.742, *p* < 0.001] Treatment [F_1,87_ = 31.036, *p* < 0.001] Epilepsy x Treatment [F_1,88_ = 17.776, *p* < 0.001]
Septo-temporal	Epilepsy [F_1,88_ = 72.050, *p* < 0.001] Treatment [F_1,88_ = 13.228, *p* < 0.001] Epilepsy x Treatment [F_1,88_ = 42.946, *p* < 0.001]
Temporal	Epilepsy [F_1,96_ = 59.882, *p* < 0.001] Treatment [F_1,96_ = 13.564. *p* < 0.001] Epilepsy x Treatment [F_1,96_ = 23.695, *p* < 0.001]
***CA2*** Septal	Epilepsy [F_1,59_ = 49.985, *p* < 0.001] Treatment [F_1,59_ = 71.588 *p* < 0.001] Epilepsy x Treatment [F_1,59_ = 59.156, *p* < 0.001]
Septo-temporal	Epilepsy [F_1,81_ = 70.550, *p* < 0.001] Treatment [F_1,81_ = 70.550, *p* < 0.001] Epilepsy x Treatment [F_1,81_ = 47.346, *p* < 0.001]
Temporal	Epilepsy [F_1,65_ = 3.531, *p* = 0.065] Treatment [F_1,65_ = 2.637, *p* = 0.109] Epilepsy x Treatment [F_1,65_ = 17.273, *p* < 0.001]
***CA3a*** Septal	Epilepsy [F_1,63_ = 7.273, *p* = 0.009] Treatment [F_1,63_ = 8.951, *p* = 0.004] Epilepsy x Treatment [F_1,63_ = 8.762, *p* = 0.004]
Septo-temporal	Epilepsy [F_1,63_ = 23.515, *p* < 0.001] Treatment [F_1,63_ = 41.089, *p* < 0.001] Epilepsy x Treatment [F_1,63_ = 26.684, *p* < 0.001]
Temporal	Epilepsy [F_1,63_ = 11.451, *p* < 0.001] Treatment [F_1,63_ = 3.851, *p* =0.053] Epilepsy x Treatment [F_1,63_ = 0.0019, *p* = 0.965]
***CA3b*** Septal	Epilepsy [F_1,90_ = 0.330, *p* = 0.567] Treatment [F_1,90_ = 0.330, *p* = 0.567] Epilepsy x Treatment [F_1,90_ = 1.356, *p* = 0.247]
Septo-temporal	Epilepsy [F_1,93_ = 4.408, *p* = 0.039] Treatment [F_1,93_ = 93.980, *p* < 0.001] Epilepsy x Treatment [F_1,93_ = 0.798, *p* = 0.374]
Temporal	Epilepsy [F_1,76_ = 8.365, *p* = 0.005] Treatment [F_1,76_ = 18.971, *p* < 0.001] Epilepsy x Treatment [F_1,76_ = 0.0003, *p* = 0.995]
***CA3c*** Septal	Epilepsy [F_1,90_ = 70.424, *p* < 0.001] Treatment [F_1,90_ = 2.969, *p* = 0.0889] Epilepsy x Treatment [F_1,90_ = 4.358, *p* = 0.039]
Septo-temporal	Epilepsy [F_1,92_ = 47.971, *p* < 0.001] Treatment [F_1,92_ = 1.371, *p* = 0.245] Epilepsy x Treatment [F_1,92_ = 0.0000566, *p* = 0.994]
Temporal	Epilepsy [F_1,92_ = 265,837; *p* < 0,001] Treatment [F_2,92_ = 17,913; *p* < 0,001] Epilepsy x Treatment [F_2,92_ = 34,335; *p* < 0,001]
***Ventral hippocampus*** ***CA1***	Epilepsy [F_1,74_ = 41.293, *p* < 0.001] Treatment [F_1,74_ = 14.954, *p* < 0.001] Epilepsy x Treatment [F_1,74_ = 15.396, *p* < 0.001]
***Ventral hippocampus*** ***CA2***	Epilepsy [F_1,93_ = 81.391, *p* < 0.001] Treatment [F_1,93_ = 19.146, *p* < 0.001] Epilepsy x Treatment [F_2,93_ = 42.975, *p* < 0.001]
***Ventral hippocampus*** ***CA3***	Epilepsy [F_1,74_ = 42.658, *p* < 0.001] Treatment [F_1,74_ = 42.658, *p* < 0.001] Epilepsy x Treatment [F_1,74_ = 64.381, *p* < 0,001]
***Hilus of the dentate gyrus*** Septal	Epilepsy [F_1,87_ = 237.156, *p* < 0.001] Treatment [F_1,87_ = 0.0386, *p* = 0.845] Epilepsy x Treatment [F_1,87_ = 0.109, *p* = 0.742]
Septo-temporal	Epilepsy [F_1,89_ = 199.646, *p* < 0.001] Treatment [F_1,89_ = 0.709, *p* = 0.402] Epilepsy x Treatment [F_1,89_ = 1.117, *p* = 0.293]
Temporal	Epilepsy [F_1,99_ = 72.320, *p* < 0.001] Treatment [F_1,99_ = 0.0920, *p* = 0.762] Epilepsy x Treatment [F_1,99_ = 1.727, *p* = 0.192]
***Dentate gyrus*** Septal	Epilepsy [F_1,91_ = 14.480, *p* < 0.001] Treatment [F_1,91_ = 0.173, *p* = 0.679] Epilepsy x Treatment [F_1,9 1_ = 0.173, *p* = 0.679]
Septo-temporal	Epilepsy [F_1,91_ = 23.872, *p* < 0.001] Treatment [F_1,91_= 0.374, *p* = 0.542] Epilepsy x Treatment [F_1,91_ = 3.047, *p* = 0.084]
Temporal	Epilepsy [F_1,93_ = 74,314; *p* < 0,001] Treatment [F_1,93_ = 1.502, *p* = 0.223] Epilepsy x Treatment [F_1,93_ = 4.562, *p* = 0.035]
***Piriform cortex*** Septal	Epilepsy [F_1,76_ = 43.071, *p* < 0.001] Treatment [F_2,76_ = 2.328, *p* = 0.131] Epilepsy x Treatment [F_2, 76_ = 8.202, *p* = 0.005]
Septo-temporal	Epilepsy [F_1,81_ = 44.779, *p* < 0.001] Treatment [F_2,81_ = 2.859, *p* = 0.095] Epilepsy x Treatment [F_2, 81_ = 2.195, *p* = 0.143]
Temporal	Epilepsy [F_1,81_ = 178.178, *p* < 0.001] Treatment [F_2,81_= 9.193, *p* = 0.004] Epilepsy x Treatment [F_1,81_ = 24.629, *p* < 0.001]
***Amygdala (basolateral)*** Septal	Epilepsy [F_1,97_ = 30.348, *p* < 0,001] Treatment [F_1,97_ = 9.565, *p* = 0.003] Epilepsy x Treatment [F_2,97_ = 17.838, *p* < 0.001]
Septo-temporal	Epilepsy [F_1,96_ = 43,690; *p* < 0,001] Treatment [F_2,96_ = 5.030, *p* = 0.0271] Epilepsy x Treatment [F_2,96_ = 28.454, *p* < 0.001]
Temporal	Epilepsy [F_1,82_ = 14.945, *p* < 0.001] Treatment [F_1,82_= 2.994, *p* = 0.087] Epilepsy x Treatment [F_2,82_ = 8.023, *p* = 0.006]

## Data Availability

Data available in a publicly accessible repository that does not issue DOIs.
